# Patterns of tau, amyloid and synuclein pathology in ageing, Alzheimer’s disease and synucleinopathies

**DOI:** 10.1093/brain/awae372

**Published:** 2024-11-12

**Authors:** Sean J Colloby, Kirsty E McAleese, Lauren Walker, Daniel Erskine, Jon B Toledo, Paul C Donaghy, Ian G McKeith, Alan J Thomas, Johannes Attems, John-Paul Taylor

**Affiliations:** Faculty of Medical Sciences, Translational and Clinical Research Institute, Newcastle University, Campus for Ageing and Vitality, Newcastle upon Tyne NE4 5PL, UK; Faculty of Medical Sciences, Translational and Clinical Research Institute, Newcastle University, Campus for Ageing and Vitality, Newcastle upon Tyne NE4 5PL, UK; Faculty of Medical Sciences, Translational and Clinical Research Institute, Newcastle University, Campus for Ageing and Vitality, Newcastle upon Tyne NE4 5PL, UK; Faculty of Medical Sciences, Translational and Clinical Research Institute, Newcastle University, Campus for Ageing and Vitality, Newcastle upon Tyne NE4 5PL, UK; Stanley H. Appel Department of Neurology, Nantz National Alzheimer Center, Houston Methodist Hospital, Houston, TX 77030, USA; Faculty of Medical Sciences, Translational and Clinical Research Institute, Newcastle University, Campus for Ageing and Vitality, Newcastle upon Tyne NE4 5PL, UK; Faculty of Medical Sciences, Translational and Clinical Research Institute, Newcastle University, Campus for Ageing and Vitality, Newcastle upon Tyne NE4 5PL, UK; Faculty of Medical Sciences, Translational and Clinical Research Institute, Newcastle University, Campus for Ageing and Vitality, Newcastle upon Tyne NE4 5PL, UK; Faculty of Medical Sciences, Translational and Clinical Research Institute, Newcastle University, Campus for Ageing and Vitality, Newcastle upon Tyne NE4 5PL, UK; Faculty of Medical Sciences, Translational and Clinical Research Institute, Newcastle University, Campus for Ageing and Vitality, Newcastle upon Tyne NE4 5PL, UK

**Keywords:** Alzheimer’s disease, dementia with Lewy bodies, Parkinson’s disease dementia, HP-tau, amyloid-β, α-synuclein

## Abstract

Alzheimer’s disease (AD) is neuropathologically defined by deposits of misfolded hyperphosphorylated tau (HP-tau) and amyloid-β. Lewy body (LB) dementia, which includes dementia with Lewy bodies (DLB) and Parkinson’s disease dementia (PDD), is characterized pathologically by α-synuclein aggregates. HP-tau and amyloid-β can also occur as co-pathologies in LB dementia, and a diagnosis of _mixed_AD/DLB can be made if present in sufficient quantities. We hypothesized that the spread of these abnormal proteins selectively affects vulnerable areas, resulting in pathologic regional covariance that differentially associates with pre-mortem clinical characteristics. Our aims were to map regional quantitative pathology (HP-tau, amyloid-β, α-synuclein) and investigate the spatial distributions from tissue microarray post-mortem samples across healthy aging, AD and LB dementia.

The study involved 159 clinico-pathologically diagnosed human post-mortem brains (48 controls, 47 AD, 25 DLB, 20 _mixed_AD/DLB, 19 PDD). The burden of HP-tau, amyloid-β and α-synuclein was quantitatively assessed in cortical and subcortical areas. Principal components (PC) analysis was applied across all cases to determine the pattern nature of HP-tau, amyloid-β and α-synuclein. Further analyses explored the relationships of these pathological patterns with cognitive and symptom variables.

Cortical (^tau^PC_1_) and temporo-limbic (^tau^PC_2_) patterns were observed for HP-tau. For amyloid-β, a cortical-subcortical pattern (^amyl^PC_1_) was identified. For α-synuclein, four patterns emerged: ‘posterior temporal-occipital’ (^syn^PC_1_), ‘anterior temporal-frontal’ (^syn^PC_2_), ‘parieto-cingulate-insula’ (^syn^PC_3_), and ‘frontostriatal-amygdala’ (^syn^PC_4_). Distinct ^syn^PC scores were apparent among DLB, _mixed_AD/DLB and PDD, and may relate to different spreading patterns of α-synuclein pathology. In dementia, cognitive measures correlated with ^tau^PC_1,_^tau^PC_2_ and ^amyl^PC_1_ pattern scores (*P* ≤ 0.02), whereas such variables did not relate to α-synuclein parameters in these or combined LB dementia cases. Mediation analysis then revealed that in the presence of ^amyl^PC_1_, ^tau^PC_1_ had a direct effect on global cognition in dementia (*n* = 65, *P* = 0.04), while ^tau^PC_1_ mediated the relationship between ^amyl^PC_1_ and cognition through the indirect pathway (^amyl^PC_1_ → ^tau^PC_1_ → global cognition) (*P* < 0.05). Last, in synucleinopathies, ^syn^PC_1_ and ^syn^PC_4_ pattern scores were associated with visual hallucinations and motor impairment, respectively (*P* = 0.02).

In conclusion, distinct patterns of α-synuclein pathology were apparent in LB dementia, which could explain some of the disease heterogeneity and differing spreading patterns among these conditions. Visual hallucinations and motor severity were associated with specific α-synuclein topographies in LB dementia that may be important to the clinical phenotype and could, after necessary testing/validation, be integrated into semiquantitative routine pathological assessment.

## Teaser

Colloby *et al.* analyse the burden and distribution of hyperphosphorylated-tau, amyloid-β and α-synuclein pathology in post-mortem brain tissue from individuals with various neurodegenerative dementias, and examine how these patterns of pathology relate to cognition and symptomatology.

## Introduction

The unifying feature of age-associated neurodegenerative diseases is the transition from physiologically soluble proteins to insoluble aggregates that accumulate and deposit in the CNS. The hallmark protein aggregates of Alzheimer’s disease (AD) are intracellular depositions of hyperphosphorylated tau (HP-tau)^1^ and extracellular amyloid-β plaques.^2^ Lewy body (LB) disease is characterized by the deposition of intracellular α-synuclein (α-syn) in the form of Lewy bodies and Lewy neurites with a typical clinical presentation of either Parkinson’s disease (PD), Parkinson's disease with dementia (PDD) or dementia with Lewy bodies (DLB). Identification of the topographical spread of α-syn can be undertaken with the Newcastle-McKeith system,^3^ recently updated to the Lewy Pathology Consensus Criteria (LPC).^4^

Hallmark protein aggregates are rarely exclusive, with ‘pure’ pathology being the exception, and frequently, more than one pathology is present, a condition referred to as cerebral multimorbidity.^5^ Cerebral multimorbidity represents a spectrum of increasing severity with clinical impact varying depending on the severity of the co-pathologies. The presence of two (or more) hallmark pathologies, each fulfilling distinct neuropathological criteria, i.e. mixed disease, is often observed at neuropathological assessment. The most common form of mixed disease is the combination of AD and limbic/neocortical α-synuclein pathology recognized as _mixed_AD/DLB^6^; where a large multicentre study showed that a mixed severe pathology was present in ∼7.5% of dementia cases, of which 72% of these had a diagnosis of _mixed_AD/DLB.^7,8^ Interestingly, 48–88% of DLB and 17–62% of PDD cases have been shown to have additional intermediate or high-level AD pathology.^9^ Concomitant AD pathology in LB dementia (DLB, PDD) also appears to drive the clinical phenotype, including earlier age of onset and a more rapid disease course.^10,11^ Given the increasing focus on disease modification, e.g. anti-amyloid and anti-tau therapies, along with emerging biologic staging systems based on biomarkers tuned to proteinopathies,^12,13^ understanding the inter-relationships and possible synergies among HP-tau, amyloid-β and α-synuclein is particularly apposite in these conditions, as this may influence future trial stratification, therapeutic decisions and patient management.

Traditional neuropathological assessment methods use limited semiquantitative staging schemes to subjectively quantify the degree of topographical spread of protein aggregates throughout the brain. Alternative approaches have now been developed to minimize assessor bias, provide objective data extraction, and enable automated methodologies for measuring HP-tau, TDP-43, amyloid-β and α-synuclein in multiple brain regions.^14-16^ In this study, we took advantage of a quantitative neuropathological technique utilizing a tissue microarray (TMA), in which a single paraffin block comprising punch biopsies from 15 distinct brain regions was composed, cut and immunohistochemically stained to allow for relatively fast, automated quantitative assessment of the pathological burden of HP-tau, amyloid-β and α-synuclein.^14^

As the brain is highly interconnected, pathologic change in one area may influence other topographically distant regions, either mediating this impact as part of co-dependent areas with selective vulnerability or based on the spread of pathology from one region to another.^17^ Additionally, distributed network dysfunction is now considered an essential component of symptoms that manifest in neurodegenerative dementias.^18^ One method of identifying patterns of connected or correlated regions of brain pathology is by principal components analysis (PCA) of the regional TMA pathology data. Therefore, we proposed that patterns of HP-tau, amyloid-β and α-synuclein in clinicopathologically diagnosed dementia groups could be investigated to identify key hubs of these specific brain pathologies, which can then be related to the clinical phenotype, particularly cognition, given the likely centrality of HP-tau and amyloid-β in driving cognitive dysfunction in AD and LB dementia.^11,19-21^

In this study, the aim was to spatially map and visualize in approximated Montreal Neurological Institute (MNI) coordinate space, regional TMA post-mortem donor tissue to investigate the pattern nature of quantified levels of HP-tau, amyloid-β and α-synuclein pathology in healthy ageing, AD, _mixed_AD/DLB, DLB and PDD. Associations with age at death, global cognition, cognitive decline, pathological staging and, where applicable, core LB dementia features (motor, cognitive fluctuations, visual hallucinations, sleep) were also examined.

## Materials and methods

### Study sample

The study comprised 159 cases obtained from Newcastle Brain Tissue Resource per approval of the joint ethics committees of Newcastle and North Tyneside Health Authority (Ref: 08/H0906/136). Patients and healthy individuals were registered into the Newcastle Brain Tissue Resource and, where appropriate, either individuals or their next of kin gave informed written consent. Most patients participated in at least one research study ante-mortem and were therefore recruited from either outpatient movement disorder or memory clinics in the Newcastle-upon-Tyne, Northumberland and Gateshead regions.

### Neuropathological processing

**Figure 1:**
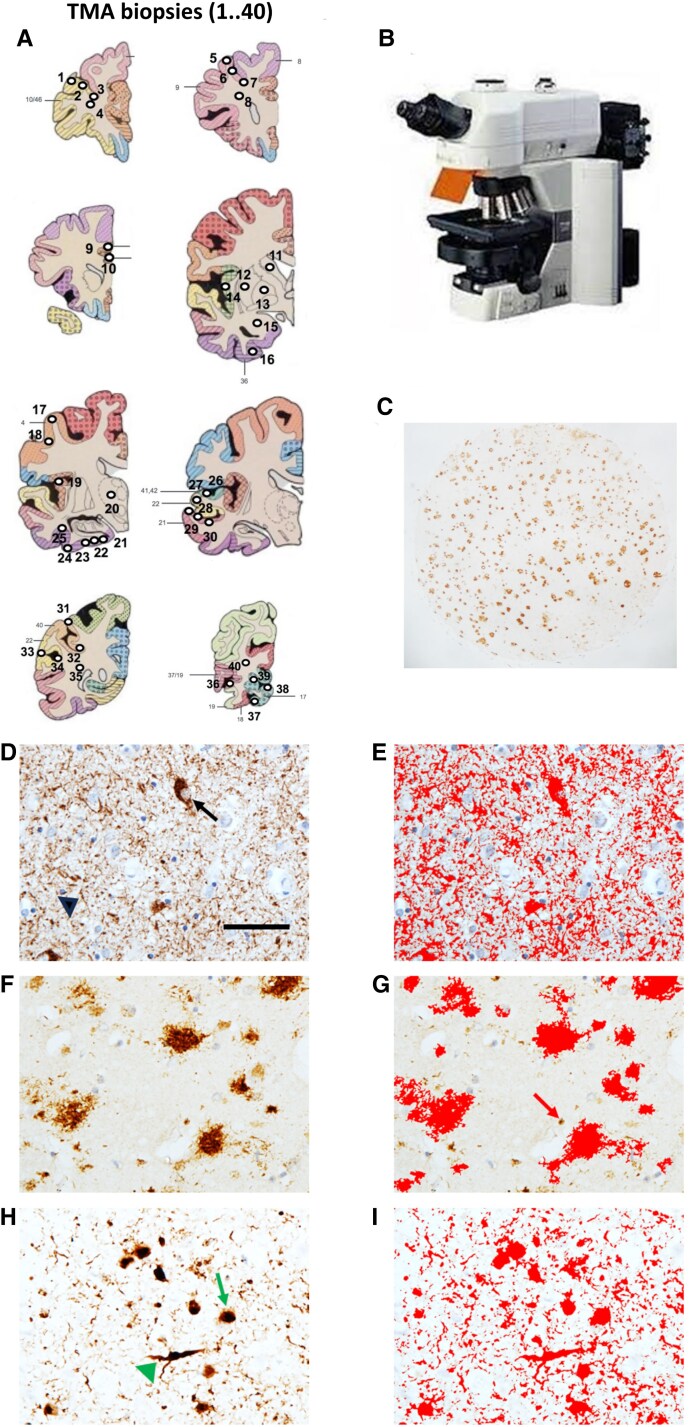
**Tissue microarray methodology**. Atlas illustration of tissue microarray (TMA) punch biopsy locations (1 → 40) (adapted from Perry and Oakley^22^) (**A**). TMA images captured on a Nikon Eclipse 90i microscope (**B**), where the whole 3 mm punch biopsy is digitally imaged (**C**). Photomicrographs illustrating immunohistochemically stained pathology for hyperphosphorylated (HP)-tau (**D**), amyloid-β (**F**) and α-synuclein (**H**), along with their corresponding applied standardized thresholds (red outlines) for the measurement of all immunopositive signals included in the quantification (**E**, **G** and **I**). In **D**, neurofibrillary tangles (arrow) and neuropil threads (arrowhead) are immunopositive for HP-tau (AT8 antibody). In **F**, plaques are immunopositive for amyloid-β (4G8 antibody), while intracellular amyloid precursor protein is also immunopositive for 4G8 but is excluded from quantification by application of a size restriction criterion (arrow) (**G**). In **H**, Lewy bodies (arrow) and Lewy neurites (arrowhead) are immunopositive for α-synuclein (α-syn antibody). Scale bar: **D** = 50 μm and is valid for all images.

At autopsy, the right hemisphere, brainstem and cerebellum were fixed in 4% formalin solution for 4 weeks. The right hemisphere was dissected into coronal slices at 7 mm intervals using a specimen cut-up system to ensure all slices were standardized and regions were then mapped according to a brain atlas (Fig. 1A; adapted from Perry and Oakley^22^). All brains underwent standardized neuropathological examinations, including Braak neurofibrillary tangles (NFT) staging,^23^ Thal amyloid phases,^2^ CERAD (Consortium to Establish a Registry for Alzheimer’s Disease) scores,^24^ NIA-AA guidelines,^25^ McKeith staging^26^ and TAR DNA-binding protein-43 (TDP-43) stages.^27^ During life, all participants undertook clinical assessments, including the Mini-Mental State Examination (MMSE).^28^ All cases had a detailed review of their clinical notes after death, with control subjects having no evidence of cognitive impairment.

Neuropathological diagnoses were based upon the degree of fulfilment of neuropathological criteria for that particular hallmark pathology. AD was defined as ‘high AD neuropathological change’, which included cases with Thal amyloid-β phase 4/5,^2^ Braak stage V/VI^1^ and CERAD stage for neuritic plaques B/C.^24^ DLB and PDD were defined as McKeith stage limbic or neocortical Lewy pathology, where a classification of DLB or PDD was established from the clinical onset of symptoms.^26^ If both ‘high AD neuropathological change’ and ‘McKeith limbic/neocortical stage’ were present, the case was labelled as _mixed_AD/DLB. Note, _mixed_AD/DLB include only DLB and not PDD cases, given PDD cases in our cohort never showed high AD pathology. In the vast majority of cases, additional low-level pathology (LowP) was apparent, though not sufficient enough to meet the criteria for diagnosing a further disease, for example, singular Braak NFT stage 0−IV or Thal Aβ phase 0–5, CERAD negative or McKeith stage amygdala and/or brainstem. In this study, we did not classify cases on the presence of LowP; however, these additional pathologies were considered part of the TMA analyses. Cases with only LowP and no clinical syndrome were assigned as controls.

Clinical and neuropathological diagnoses were combined to state a clinicopathological diagnosis. Thus, the study cohort consisted of 48 controls, 47 AD, 25 DLB, 20 _mixed_AD/DLB and 19 PDD. One hundred and seven cases had at least one MMSE assessment before death [test-to-death interval (years): mean = 1.9, standard deviation (SD) = 1.7] (Table 1) and an estimate of the annual rate of change in MMSE (ΔMMSE/year) was available from repeated measures in 38 cases (*n* = 3 controls, *n* = 8 AD, *n* = 11 DLB, *n* = 5 _mixed_AD/DLB, *n* = 11 PDD). The latter, assuming a linear change, was calculated as the difference between the last and earliest MMSE scores divided by the duration in years between these assessments (Table 1).

**Table 1:** Study characteristics (*n* = 159)

	Controls	AD	DLB	_mixed_AD/DLB	PDD	Statistic, *P*-value
*n*	48	47	25	20	19	–
Sex (m: f)	21: 27	24: 23	18: 7	14:6	14: 5	**χ^2^ = 11.3, 0.02**
NFT Braak stages (0:I–II:III–IV:V–VI)	7:25:15:1	0:0:2:45	0:10:12:3	0:0:1:19	2:7:10:0	**χ^2^ = 158.3, < 0.001**
CERAD scores (neg:A:B:C)	39:4:4:1	0:0:2:45	11:5:6:3	0:0:1:19	13:3:3:0	**χ^2^ = 148.8, < 0.001**
Thal phases^a^ (0:1:2:3:4:5)	15:9:8:5:5:4	0:0:0:1:5:38	2:2:0:5:3:1	0:0:0:1:2:15	0:1:1:3:2:2	**χ^2^ = 104.8, < 0.001**
NIA-AA^b^ (none:1:2:3)	15:22:7:1	0:0:2:42	2:6:5:0	0:0:1:15	0:6:3:0	**χ^2^ = 126.1, < 0.001**
McKeith^c^ (0:1:2:3:other^d^)	48:0:0:0:0	40:0:0:0:7	1:0:4:18:0	0:0:1:17:0	0:0:4:15:0	**χ^2^ = 172.2, < 0.001**
TDP-43^e^ (0:1:2:3:4:5:6)	41:0:1:2:2:0:0	20:6:2:3:10:1:1	10:0:0:0:1:1:0	7:1:1:4:2:0:0	7:2:0:0:0:0:0	**χ^2^ = 43.4, 0.009**
APOE4 allele^f^ (absent: present)	26: 8	13: 19	7: 10	3: 11	6: 7	**χ^2^ = 15.8, 0.003**
Age at death	82.1 ± 11.3	84.0 ± 8.1	77.7 ± 6.2	78.5 ± 6.3	77.4 ± 6.7	*W*(4,66.7) = 5.0, 0.001*
Final MMSE^g^	27.9 ± 2.3	9.6 ± 8.7	13.2 ± 8.0	8.5 ± 7.9	16.0 ± 8.9	*H*(4) = 51.4, < 0.001**
Test-to-death interval (years)^g^	1.4 ± 1.5	2.1 ± 1.9	1.7 ± 1.5	2.3 ± 1.9	1.8 ± 1.5	*H*(4) = 3.1, 0.6
ΔMMSE_avg_ (yr^−1^)^h^	−0.4 ± 0.3	−3.4 ± 1.8	−2.7 ± 2.2	−4.3 ± 3.2	−3.5 ± 3.9	*H*(4) = 5.7, 0.2
Interval (first and last MMSE, years)^h^	7.6 ± 4.1	4.3 ± 1.7	2.7 ± 1.9	2.8 ± 2.0	2.7 ± 1.9	*H*(4) = 9.3, 0.06
UPDRS III^i^	–	–	43.9 ± 19.0	30.2 ± 18.9	39.0 ± 11.7	*F*(2,15) = 1.0, 0.4
CAF^j^	–	–	10.1 ± 3.6	6.3 ± 2.1	9.8 ± 2.0	*F*(2,14) = 2.5, 0.1
NPI_hall_^j^	–	–	4.6 ± 4.3	2.5 ± 3.0	3.4 ± 3.4	*F*(2,14) = 0.5, 0.6
NPI_sleep_^j^	–	–	1.5 ± 2.1	2.3 ± 2.9	2.4 ± 5.4	*H*(2) = 0.4, 0.8

Values denote mean ± 1 standard deviation. neg = negligible. Bold text denotes statistical significance. AD = Alzheimer’s disease; CAF = cognitive fluctuations score; CERAD = Consortium to Establish a Registry for Alzheimer’s Disease; DLB = dementia with Lewy bodies; f = female; m = male; MMSE = Mini-Mental State Examination; ΔMMSE_avg_ = estimate of average annual rate of change in MMSE; NIA-AA = National Institute on Aging and the Alzheimer’s Association; NFT = neurofibrillary tangles; NPI_hall_, NPI_sleep_ = hallucinations and sleep subdomains of the Neuropsychiatric Inventory; PDD = Parkinson’s disease dementia; UPDRS = Unified Parkinson’s Disease Rating Scale.

Incomplete data:

^a^ *n* = 130.

^b^ *n* = 127.

^c^ *n* = 155.

^d^McKeith ‘other’ refers to unclassifiable cases showing α-synuclein pathology only in the amygdala or olfactory bulb/tract.

^e^ *n* = 125.

^f^ *n* = 110.

^g^Con *n* = 20, AD *n* = 36, DLB *n* = 18, _mixed_AD/DLB *n* = 17, PDD *n* = 16.

^h^Con *n* = 3, AD *n* = 8, DLB *n* = 11, _mixed_AD/DLB *n* = 5, PDD *n* = 11.

^i^DLB *n* = 8, _mixed_AD/DLB *n* = 5, PDD *n* = 5.

^j^DLB *n* = 8, _mixed_AD/DLB *n* = 4, PDD *n* = 5.

*Post hoc* tests:

*AD > DLB, AD/DLB, PDD (*P* ≤ 0.03); otherwise not significant (Games-Howell).

**Con > AD, DLB, AD/DLB, PDD (*P* ≤ 0.004), otherwise not significant (Mann-Whitney U).

### Quantification

For each case, a TMA block containing a regionally distributed set of 40 3-mm diameter core punch biopsies (1 → 40) were obtained (Fig. 1A). The 40 punches were taken from the following areas: pre-frontal [Brodmann area (BA) 9, 10/46], mid-frontal (BA8, 9), cingulate (BA24, 32), caudate, putamen, external globus pallidus, amygdala, insula, precentral (BA4), thalamus, entorhinal cortex, temporal (BA21, 22, 41/42), parietal (BA 40) and occipital cortices (BA17, 18, 19, 19/37).^14^

Sections (6 μm) were then cut and immunohistochemically stained for HP-tau (AT8 clone, dilution 1:4000, Innogenetics, Belgium) (Fig. 1D), amyloid-β (4G8 clone, dilution 1:15 000, Covance) (Fig. 1F) and α-synuclein (KM51, dilution 1:200, Leica) (Fig. 1H) as previously described.^14,29^ A Nikon Eclipse 90i microscope (Fig. 1B) coupled to NIS Elements software v 3.0 (Nikon), where each of the 40 tissue samples have 3 × 3 single images captured at 100× magnification to create a combined image of 1.7 mm^2^. If necessary, cases were also subjected to manual setting of regions of interest to exclude meningeal structures, large vessels or white matter. Standardized thresholds for immunopositivity, set to the RGB intensity values for binary layer pixels, were applied separately for AT8 (Fig. 1E), 4G8 (Fig. 1G) and α-synuclein (Fig. 1I) immunoreactivity, in addition to a size restriction threshold of over 100 μm^2^ for the assessment of 4G8 to exclude the measurement of immunoreactive signals from physiological amyloid precursor protein (APP) (Fig. 1G). The percentage areas covered by AT8, 4G8 and α-synuclein immunoreactivity were then obtained for each sample, providing regional (R_1_ → R_40_) quantitative measurements of the pathologic load for HP-tau, amyloid-β and α-synuclein. This approach contrasts with many other studies that report only the presence or absence, or a subjective semiquantitative score, i.e. mild, moderate, severe, of these pathologies.

### Approximated spatial mapping to MNI space

**Figure 2:**
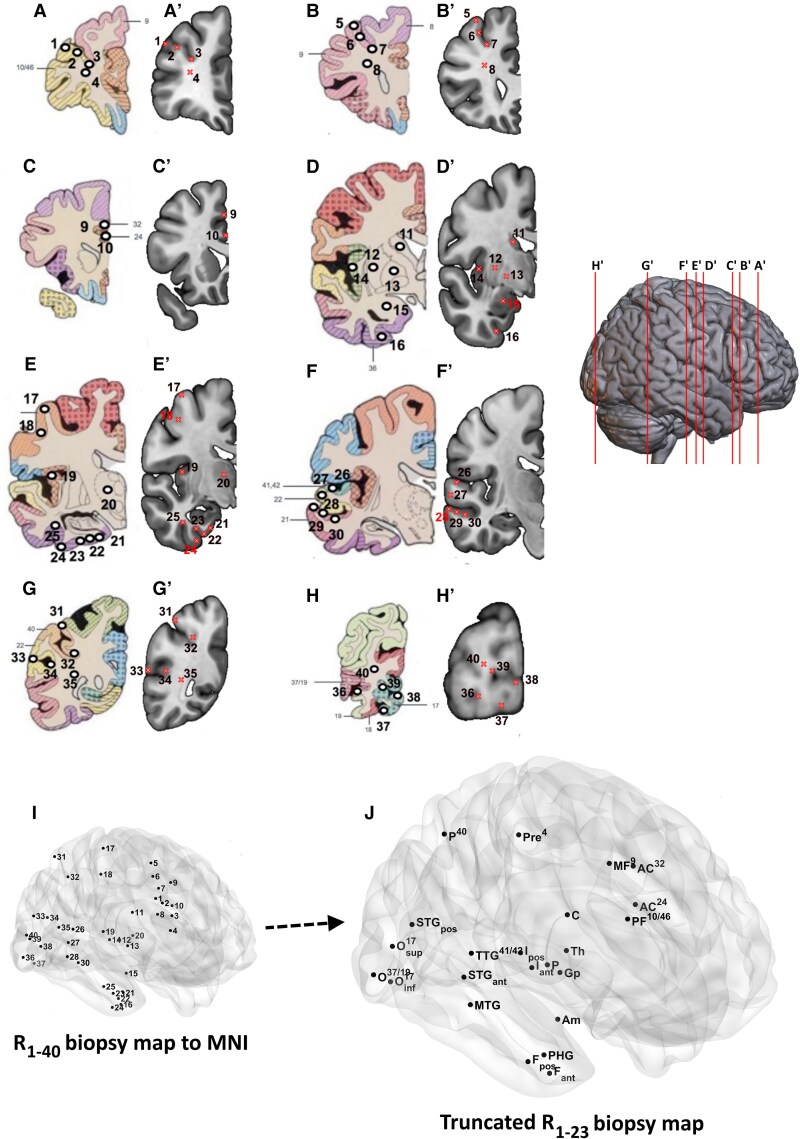
**Tissue microarray spatial mapping**. Atlas drawing of each of the tissue microarray (TMA) cores extracted with numerical labels (**A** → **H**) and their approximated mappings to the 152_MNI T_1_-weighted MRI brain template (**A’** → **H’**). Images showing TMA punch biopsies mapped to estimated MNI space (**I**), along with the truncated set (**J**).

The atlas illustration that defined the location of each punch biopsy (R_1_ → R_40_) was then approximately mapped onto coronal sections of a 152_MNI T_1_-weighted MRI brain template (right hemisphere). Figure 2 represents the alignment of each block (A → H) to corresponding sections of the MNI brain template (A’ → H’). Figure 2I depicts each regional biopsy superimposed onto a surface-rendered MNI brain template image, i.e. the ‘R_1_–_40_ biopsy map’. Supplementary Table 1 details each punch biopsy (1 → 40) along with their approximate MNI coordinates and associated annotations. Further adjustments to the R_1_–_40_ biopsy map were then undertaken to take into account punch biopsies of proximal cytoarchitecture, by averaging selected brain regions in terms of coordinates and related quantifications. This generated a truncated ‘R_1_–_23_ biopsy map’ (Fig. 2J and Supplementary Table 2), from which all subsequent data analyses were based. Supplementary Table 3 details the percentage area [mean ± standard error (SE)] of the tissue covered by tau, amyloid-β and α-synuclein immunopositivity for each region (R_1_–_23_) across all ageing and dementia cases. Region maps and pathologic patterns were displayed with the brain network visualization tool ‘BrainNet Viewer’ (http://www.nitrc.org/projects/bnv/).

### Statistical analyses

Analysis used IBM SPSS v. 27.0 and R (v. 4.2.0, https://www.R-project.org/). Demographic and behavioural variables were tested for normality and variance homogeneity using Shapiro-Wilk and Levene’s tests, respectively. Where applicable, data were examined with parametric (ANOVA *F*, Welch’s ANOVA *W*, Pearson’s *r*) and non-parametric (Kruskal-Wallis *H*, χ^2^, full and partial Spearman’s ρ) tests. *Post hoc* procedures utilized either Games-Howel or Mann-Whitney U-tests. For correlations, Benjamini-Hochberg *P*-value adjustment for multiple comparisons were applied to reduce the risk of type I errors (P´).

PCA was applied to the truncated TMA dataset (R_1_ → R_23_) across all cases simultaneously. PCA combines a set of correlated variables into a reduced number of parameters (principal components, PCs), such that the first few retain most of the original data variance. Variables with a PC loading ≥ 0.6 were considered as having a significant contribution.^30,31^ The number of extracted PCs was determined from ‘parallel analyses’, where PC eigenvalues that exceed those generated from simulated data of equal size were retained. PC rotation was implemented to achieve ‘simple structure’, ensuring each variable associates strongly with only one PC. In the present study, the oblimin procedure was adopted, allowing—if they existed—correlations between PCs. For each extracted PC, pattern scores of all cases were obtained and standardized (μ = 0, σ = 1), where higher values denoted greater case expressions of that PC. For each PC, average pattern scores were then calculated in controls, AD, _mixed_AD/DLB, DLB and PDD. Each PC represented a pattern or grouping of strongly connected regions of brain pathology. In addition, Kaiser-Meyer-Olkin measures of sampling adequacy and Bartlett’s tests of sphericity supported data suitability for PCA, respectively (0.73–0.94; 2334.5 ≤ χ^2^ ≤ 6045.9, df = 253, *P* < 0.001).

Mediation analysis was used to understand the various pathways through which an independent/predictor variable influences a dependent/outcome measure. Specifically here, we addressed how pathological pattern scores related to measures of cognition directly, as well as indirectly through interactions with other brain pathologies. The effect of an independent variable on an outcome is partitioned into direct and indirect effects. The direct impact is the relationship between the independent and outcome in the presence of any mediators and other covariates, whilst the indirect effect is the product of the unique effect of the independent on the mediator with the unique effect of the mediator on the outcome. Thus, the indirect effect measures the extent by which the independent → outcome relationship is transmitted or mediated through another variable. All analyses used ‘model 4’ SPSS ‘PROCESS’ macro v. 4.1 (https://www.processmacro.org/index.html), with 10 000 bootstrap samples for percentile bootstrap confidence intervals.

## Results

### Subjects

Table 1 shows demographic and clinical characteristics of the study population. As expected, the proportion of males to females was greater in DLB, _mixed_AD/DLB and PDD than in controls and AD. Age at death differed between groups, with AD cases being older than DLB and PDD. MMSE scores closest to death were higher in controls compared to disease groups but similar among patients. Categorical neuropathology staging scores were significantly greater in the dementia subgroups relative to controls.

### Patterns of hyperphosphorylated-tau, amyloid-β and α-synuclein

**Figure 3:**
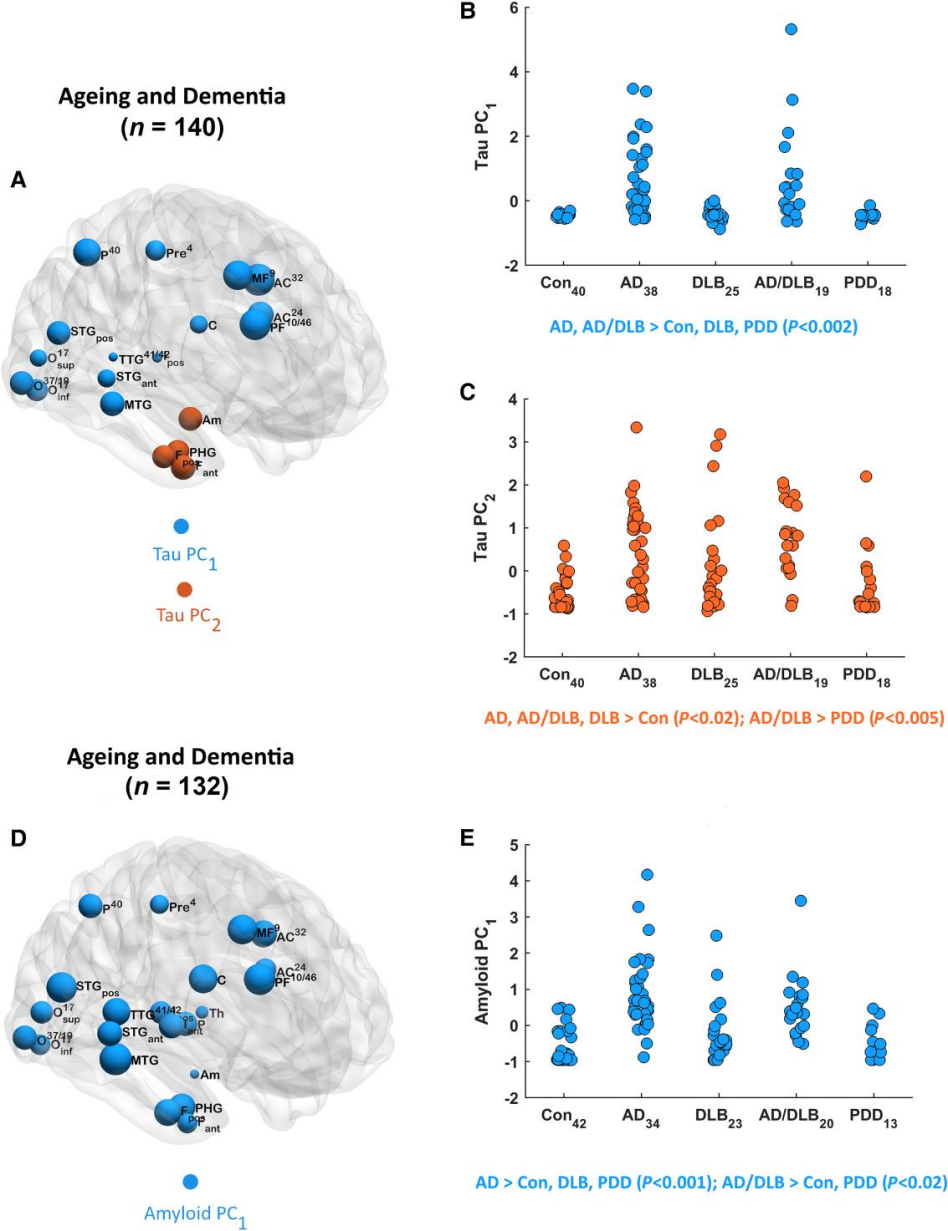
**Principal components analysis results**. Quantitative tau (**A**), where regions/nodes significantly contributing to each principal component (PC) are shown (node size proportional to PC loadings). Plots of ^tau^PC scores (standardized) against diagnosis (**B** and **C**). Quantitative amyloid-β (**D**), with regions/nodes significantly contributing to the PC. Plot of ^amyl^PC_1_ scores (standardized) against diagnosis (**E**). AD = Alzheimer’s disease; Con = controls; DLB = dementia with Lewy bodies; PCA = principal components analysis; PDD = Parkinson’s disease dementia.

For HP-tau pathology, two PCs emerged (Supplementary Table 4) across all cases (dementia and controls) (*n* = 140), accounting for 61.9% of the total original data variance. The first (^tau^PC_1_, 53% variance) comprised frontal, parietal, occipital and anterior cingulate regions, suggesting a distributed cortical pattern. The second (^tau^PC_2_, 8.9% variance) involved an inferior temporal limbic pattern confined to the temporal lobe, specifically the amygdala, anterior/posterior fusiform and parahippocampus. Figure 3A illustrates ^tau^PC_1_ (blue) and ^tau^PC_2_ (orange) patterns with contributing regions as well as corresponding plots of ^tau^PC scores (standardized) as a function of diagnosis (Fig. 3B and C). Table 2 shows average ^tau^PC scores across groups, where—as expected—^tau^PC_1_ values were significantly higher in AD and _mixed_AD/DLB compared to DLB, PDD and controls. For ^tau^PC_2_, scores were significantly greater in AD, ^mixed^AD/DLB and DLB relative to controls, and ^mixed^AD/DLB relative to PDD.

**Table 2:** Pathological principal component pattern scores stratified by clinicopathological diagnosis

	Controls	ad	DLB	_mixed_AD/DLB	PDD	Statistic, *P*-value
Tau PC_1_	−0.45 ± 0.04	0.54 ± 1.11	−0.26 ± 0.91	0.66 ± 1.50	−0.47 ± 0.12	***H*(4) = 57.2, < 0.001^a^**
Tau PC_2_	−0.60 ± 0.35	0.35 ± 1.10	0.08 ± 1.18	0.75 ± 0.85	−0.31 ± 0.79	***H*(4) = 41.6, < 0.001^b^**
Amyloid PC_1_	−0.70 ± 0.45	0.88 ± 1.04	−0.21 ± 0.81	0.52 ± 0.86	−0.48 ± 0.49	***H*(4) = 69.9, < 0.001^c^**
Synuclein PC_1_	−0.24 ± 0.0008	−0.25 ± 0.04	0.75 ± 2.16	0.16 ± 1.01	0.08 ± 0.59	*H*(4) = 6.9, 0.14
Synuclein PC_2_	−0.26 ± 0.001	−0.25 ± 0.06	0.23 ± 0.85	0.92 ± 2.49	0.06 ± 0.49	***H*(4) = 89.2, < 0.001^d^**
Synuclein PC_3_	−0.17 ± 0.001	−0.18 ± 0.004	0.11 ± 0.50	0.66 ± 2.75	0.07 ± 0.40	***H*(4) = 40.8, < 0.001^d^**
Synuclein PC_4_	−0.43 ± 0.005	−0.39 ± 0.15	0.84 ± 1.51	0.27 ± 1.25	0.69 ± 1.27	***H*(4) = 76.1, < 0.001^d^**

Values denote (mean ± standard deviation) of pattern scores for each principal component (PC). Bold text denotes statistical significance (*P* < 0.05).

Group sizes *n*: Tau = Control (Con) 40, Alzheimer's disease (AD) 38, dementia with Lewy bodies (DLB) 25, AD/DLB 19, Parkinson's disease dementia (PDD) 18; Amyloid = Con 42, AD 34, DLB 23, AD/DLB 20, PDD 13; Synuclein = Con 48, AD 47, DLB 25, AD/DLB 19, PDD 19. *Post hoc* Mann-Whitney U-tests (Bonferroni adjusted for multiple comparisons).

^a^AD, AD/DLB > Con, DLB, PDD (*P* ≤ 0.002); otherwise not significant.

^b^AD > Con (*P* < 0.001); AD/DLB > Con, PDD (*P* ≤ 0.005); DLB > Con (*P* = 0.02); otherwise not significant.

^c^AD > Con, DLB, PDD (*P* ≤ 0.001); AD/DLB > Con, PDD (*P* ≤ 0.02); otherwise not significant.

^d^AD/DLB, DLB, PDD > Con, AD (*P* < 0.01); otherwise not significant.

For amyloid-β pathology, the analysis revealed one PC across all cases (*n* = 132). Supplementary Table 4 depicts the solution (^amyl^PC_1_), accounting for 70.1% of the total variance of the original data, where ^amyl^PC_1_ consisted of all but one of the regions, indicating a widely distributed cortical-subcortical global brain pattern. Figure 3D shows ^amyl^PC_1_ (blue) and contributing regions, as well as a graph of ^amyl^PC_1_ scores (standardized) against diagnosis (Fig. 3E). Table 2 displays the average ^amyl^PC_1_ scores across groups, with values significantly higher in AD and _mixed_AD/DLB relative to controls and PDD, as well as AD compared to DLB.

**Figure 4:**
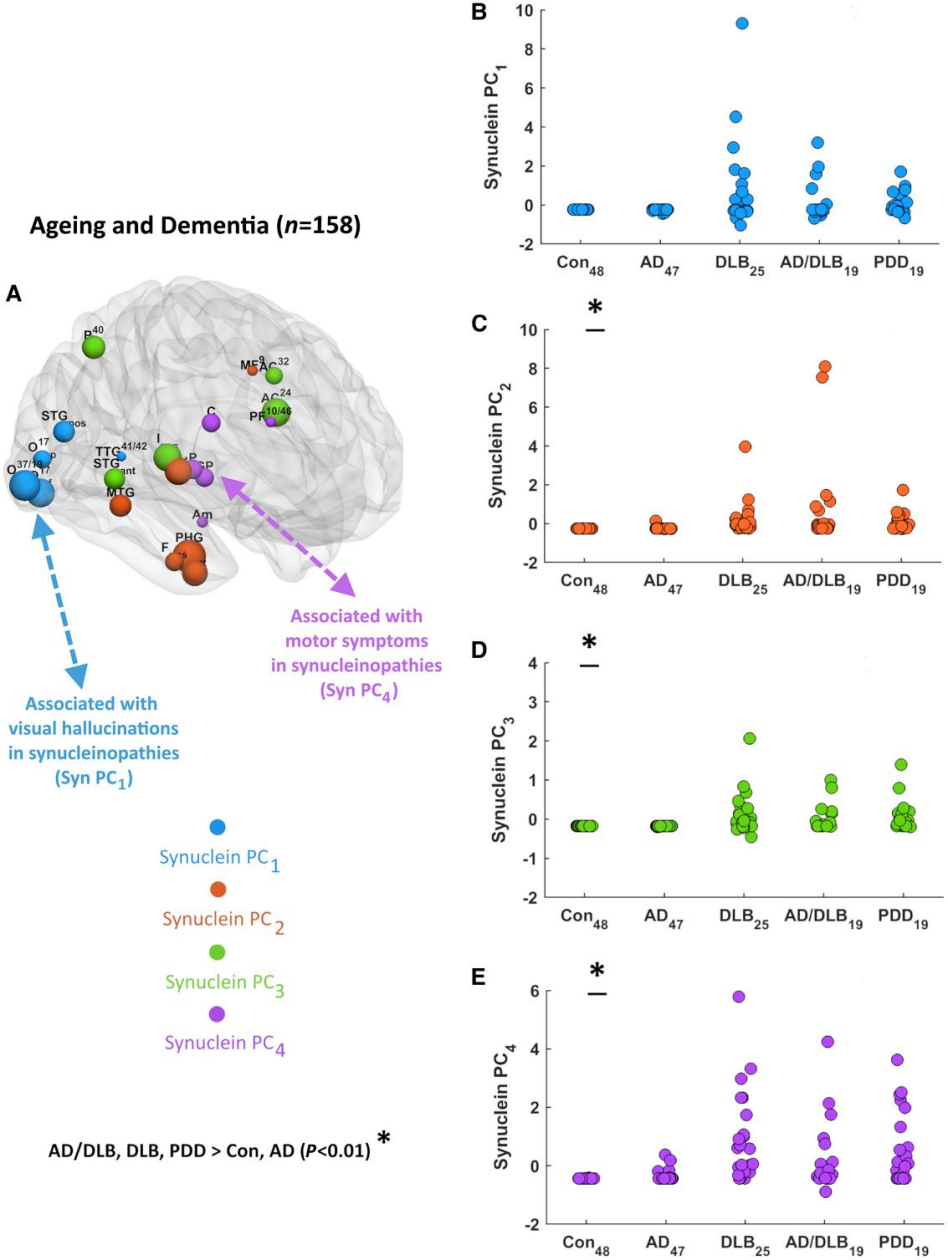
**Principal components analysis results**. Quantitative α-synuclein (**A**), where regions/nodes significantly contributing to each principal component (PC) are shown (node size proportional to PC loadings). Plots of each ^syn^PC score (standardized) against diagnosis (**B**–**E**). AD = Alzheimer’s disease; Con = controls; DLB = dementia with Lewy bodies; PCA = principal components analysis; PDD = Parkinson’s disease dementia.

For α-synuclein pathology, four PCs were extracted (Supplementary Table 4) across all cases (*n* = 158), accounting for 79% of the total original data variance. The first (^syn^PC_1_, 44% variance) contained transverse and superior temporal gyri and structures within the occipital lobe (37/19, 17_inf_, 17_sup_) consistent with a posterior temporo-occipital pattern. The second (^syn^PC_2_, 15.3% variance) involved the anterior insula, temporal lobe structures (fusiform, parahippocampus, middle temporal gyrus) and medial frontal, suggesting a frontal-anterior temporal pattern. The third (^syn^PC_3_, 10.8% variance) consisted of the anterior cingulate (24, 32), posterior insula, superior temporal gyrus (anterior aspect) and parietal lobe, which defined a temporoparietal-insulo-cingulate pattern. Last, (^syn^PC_4_, 8.9% variance) included the caudate, putamen, globus pallidus, amygdala and prefrontal regions, indicating a frontostriatal-amygdala pattern. Figure 4A displays each α-synuclein pattern (^syn^PC_1 ‘_blue’, ^syn^PC_2 ‘_orange’, ^syn^PC_3 ‘_green’, ^syn^PC_4 ‘_purple’), their contributing regions, associated ^syn^PC scores (standardized) and plotted against diagnosis (Fig. 4B–E, respectively). Table 2 shows the average ^syn^PC scores across groups, with ^syn^PC_2_, ^syn^PC_3_ and ^syn^PC_4_, as expected, significantly higher in _mixed_AD/DLB, DLB and PDD than AD and controls. For ^syn^PC_1_, scores did not differ between groups (*P* > 0.05).

*APOE* ε4 data were also available for a large proportion of cases in the cohort (*n* = 110; Table 1). Supplementary Table 5 represents the average pathological PC pattern scores for each group stratified by, where applicable, *APOE* ε4 status (absent, present). Statistical analyses revealed that across all cases, significant correlations were observed for ^tau^PC_2_ (ρ = 0.35, *P*´ < 0.001)^*n* = 95^ and ^amyl^PC_1_ (ρ = 0.51, *P*´ < 0.001)^*n* = 89^ but not ^tau^PC_1_ (ρ = 0.15, *P*´ = 0.15)^*n* = 95^ nor ^syn^PC_1,23,4_ (|ρ| ≤ 0.12, *P*´ ≥ 0.52)^*n* = 109^ with APOE4 status. On the individual group level, APOE4 status was found not to be associated with ^tau^PC_1,2_ (|ρ| ≤ 0.42, *P* ≥ 0.09), ^amyl^PC_1_ (|ρ| ≤ 0.68, *P* ≥ 0.10) or ^syn^PC_1,23,4_ (|ρ| ≤ 0.45, *P* ≥ 0.12) pattern scores. This suggests a minimal effect, although the lack of such correlations observed were likely attributed to the relatively small and unbalanced representation of APOE4 data within each group.

**Figure 5:**
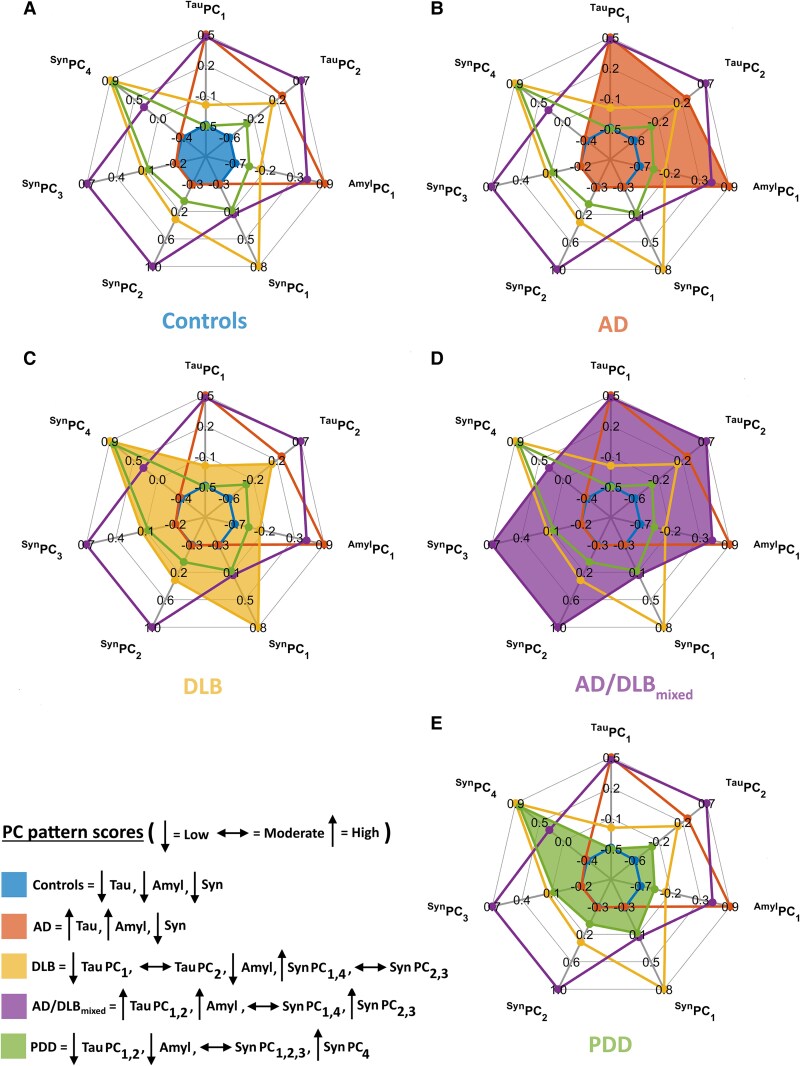
**Radar plots of the pathological patterns**. Summarizing the average scores (‘low’ < 0, 0 ≤ ‘moderate’ ≤ 0.2, ‘high’ > 0.2) for each diagnostic group (shaded regions, **A**–**E**). AD = Alzheimer’s disease; DLB = dementia with Lewy bodies; PC = principal component; PDD = Parkinson’s disease dementia.

Figure 5 utilizes radar plots to summarize for each diagnostic group, mean values of HP-tau, amyloid-β and α-synuclein PC pattern scores (‘low’ < 0, 0 ≤ ‘moderate’ ≤ 0.2, ‘high’ > 0.2; Controls *n* = 37, AD *n* = 32, DLB *n* = 21, _mixed_AD/DLB *n* = 17, PDD *n* = 11). Controls, AD and DLB (Fig. 5A–C) demonstrated scores that were typically indicative of their corresponding neuropathological definitions, i.e. Con (low ^tau^PC_1,2_, low ^amyl^PC_1_, low ^syn^PC_1,23,4_), AD (high ^tau^PC_1,2_, high ^amyl^PC_1_, low ^syn^PC_1,23,4_) and DLB (low ^tau^PC_1_, moderate ^tau^PC_2_, low ^amyl^PC_1_, high ^syn^PC_1,4_, moderate ^syn^PC_2,3_). In _mixed_AD/DLB, high ^tau^PC_1,2_, high ^amyl^PC_1_, moderate ^syn^PC_1,4_ and high ^syn^PC_2,3_ were observed (Fig. 5D), while PDD exhibited low ^tau^PC_1,2_, low ^amyl^PC_1_, moderate ^syn^PC_1,2,3_ and high ^syn^PC_4_ (Fig. 5E). Notably, each group with significant LB pathology (DLB, _mixed_AD/DLB, PDD), showed distinct ^syn^PC profiles, which may relate to symptomatic heterogeneity and differing spreading patterns of α-synuclein pathology among these conditions.

### Relationship between pattern scores and cognition

Association between pathological pattern scores with age at death, final MMSE and average annual rate of change in MMSE (ΔMMSE_avg_) were investigated in the combined dementia cohort (AD, DLB, _mixed_AD/DLB, PDD). As some deviation was apparent in the duration between first and last MMSE assessments across groups, relationships involving ΔMMSE_avg_ were examined with partial correlations (ρ^partial^, adjusted for time interval between MMSEs). First, final MMSE was inversely correlated with ^tau^PC_1_ (ρ = −0.33, *P*´ = 0.004)^*n* = 79^ while ΔMMSE_avg_ was inversely related to ^tau^PC_2_ (ρ^partial^ = −0.40, *P*´ = 0.04)^*n* = 30^. Second, ^amyl^PC_1_ score was associated with age at death (ρ = 0.26, *P*´ = 0.01)^*n* = 90^, final MMSE (ρ = −0.25, *P*´ = 0.02)^*n* = 70^ and ΔMMSE_avg_ (ρ^partial^ = −0.39, *P*´ = 0.02)*^n^*^= 30^. Third, α-synuclein pattern scores (^syn^PC_2_, ^syn^PC_3,_ and ^syn^PC_4_) correlated with age at death (−0.38 ≤ ρ ≤ −0.19, *P*´ ≤ 0.03)^*n* = 110^. All other correlations were non-significant (*P*´ > 0.05). In addition, we further looked into the relationship between cognition and all four α-synuclein pattern scores in a merged group of cases with significant LB pathology (DLB, _mixed_AD/DLB, PDD), where no associations were observed with MMSE (|ρ| ≤ 0.24, *P*´ ≥ 0.16)^*n* = 57^ or ΔMMSE_avg_ (|ρ| ≤ 0.22, *P*´ ≥ 0.34)^*n* = 28^.

### Mediation analyses

**Figure 6:**
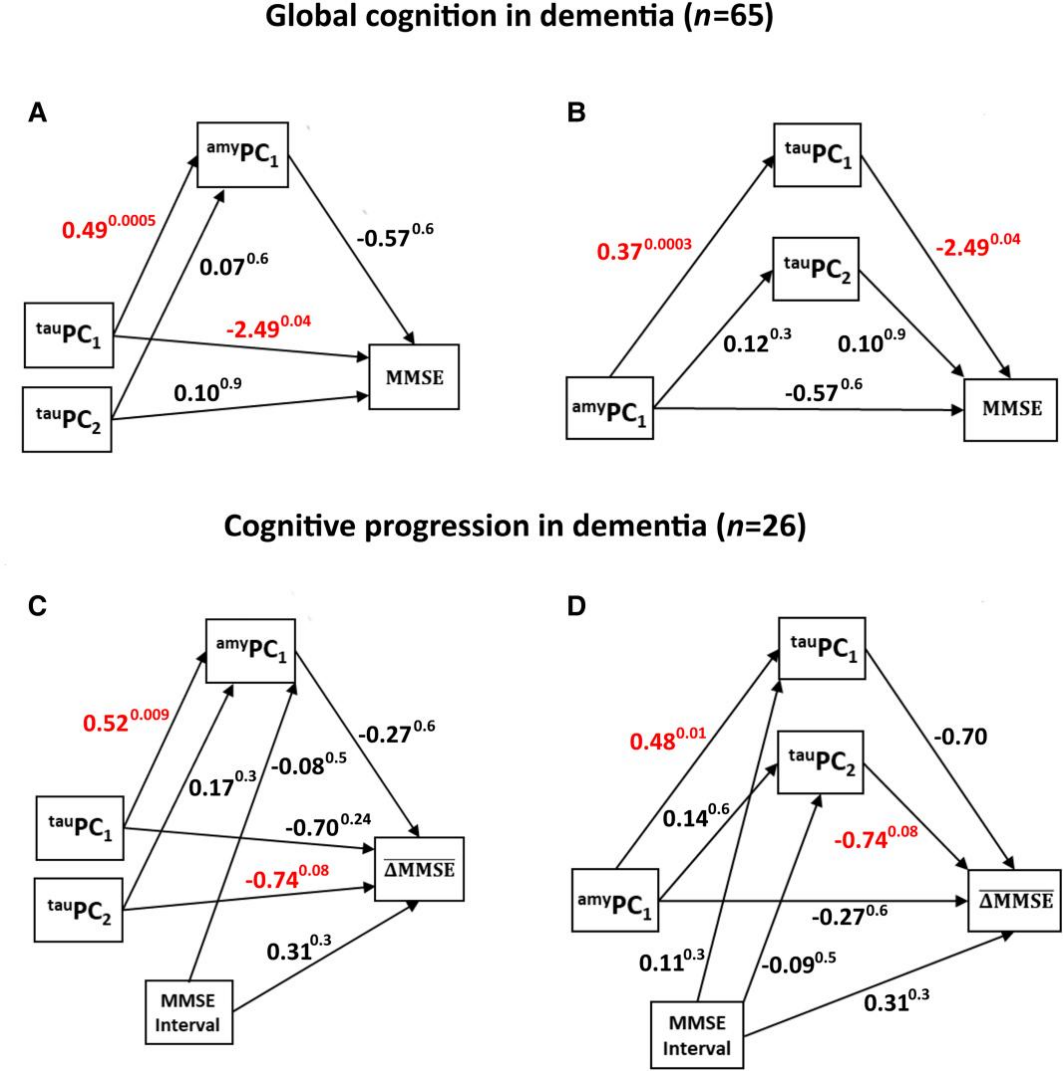
**Mediation modelling**. Relationship of neuropathological principal component (PC) pattern scores with global cognition (**A** and **B**) and progression (**C** and **D**) in dementia. Values denote unstandardized regression coefficients β with corresponding *P*-values (β^p−value^). MMSE = Mini-Mental State Examination.

As HP-tau and amyloid-β PC pattern scores (but not α-synuclein) correlated with cognitive measures in dementia cases, we then further examined the inter-relationship of tau and amyloid on cognition, or more specifically, the role of HP-tau in the presence of amyloid-β and vice versa on cognitive outcomes. To test this, we applied two mediation models, where analyses were confined to dementia cases to remove non-dementia/dementia group effects from confounding the results. We did not control for the test-to-death interval or age at death in any model as neither of these variables correlated with any of the outcome measures, i.e. MMSE (|r|≤0.08, *P* ≥ 0.6) and ΔMMSE_avg_ (|r|≤0.2, *P* ≥ 0.3). However, some variation was apparent in the duration between first and last MMSE assessments across dementia groups for the calculation of ΔMMSE_avg_, and therefore was included as an additional covariate to the respective models. The first model examined the mediating effect of amyloid-β (^amyl^PC_1_) on the relationship of each HP-tau PC (^tau^PC_1,_^tau^PC_2_) on cognition, while the second assessed the mediating effects of ^tau^PC_1_ and ^tau^PC_2_ on the relationship with ^amyl^PC_1_ on similar outcomes. Figure 6A and B depict the first and second models, respectively, their unstandardized regression coefficients and corresponding *P*-values (β^p−value^), with MMSE as the dependent variable. The first mode1 revealed that, in the presence of ^amyl^PC_1_, ^tau^PC_1_ had a direct effect on MMSE (*P* = 0.04), but no significant indirect effect (IE) of ^tau^PC_1_ or ^tau^PC_2_ on MMSE through ^amyl^PC_1_ [IE_tau_pc1_ = −0.28, 95% confidence interval (CI): −1.41 to 0.99; IE_tau_pc2_ = −0.04; 95% CI: −0.39 to 0.38]. The second model showed, in the presence of ^tau^PC_1_ and ^tau^PC_2_, ^amyl^PC_1_ had no direct effect on MMSE (*P* = 0.6), however, a significant indirect effect was observed between ^amyl^PC_1_ and MMSE via ^tau^PC_1_ (IE = −0.93; 95% CI: −2.25 to −0.23) but not ^tau^PC_2_ (IE = 0.01; 95% CI: −0.53 to 0.39). Hence, ^amyl^PC_1_ does not mediate the relationships of either ^tau^PC_1_ or ^tau^PC_2_ on MMSE, whereas ^tau^PC_1_ fully mediated the relationship between ^amyl^PC_1_ and MMSE. This implies that the effect of ^tau^PC_1_ on MMSE was direct, while the effect of ^amyl^PC_1_ on MMSE was indirect through its impact on ^tau^PC_1_, i.e. ^amyl^PC_1_ → ^tau^PC_1_ → MMSE. Figure 6C and D depict mediation models for ΔMMSE_avg_ (global cognitive decline) as the outcome measure. No significant direct or indirect effects were found for either model, albeit there was a trend, whilst holding the effects of ^amyl^PC_1_, ^tau^PC_1_ and MMSE interval constant, of ^tau^PC_2_ on progression (*P* = 0.08).

### Relationship between pattern scores and pathological staging

As proof-of-concept, the association of each pathological pattern score and their corresponding pathological staging (Braak NFT-tau, Thal amyloid phases, McKeith α-synuclein) were also examined in the combined dementia cohort. Braak NFT staging correlated with ^tau^PC_1_ (ρ = 0.68, *P*´ < 0.001)^*n* = 100^ and ^tau^PC_2_ (ρ = 0.34, *P*´ < 0.001)^*n* = 100^, Thal phases with ^amyl^PC_1_ (ρ = 0.56, *P*´ < 0.001)^*n* = 67^, and McKeith staging with ^syn^PC_2_, ^syn^PC_3,_ and ^syn^PC_4_ (0.35 ≤ ρ ≤ 0.52, *P*´ < 0.001)^*n* = 107^.

### Relationship between pattern scores and Lewy body symptoms

Within the group of cases with significant LB pathology (DLB, _mixed_AD/DLB, PDD) with complete data (*n* = 49), we undertook an exploratory approach to study the relationship of each of the seven neuropathological pattern scores with pre-mortem measures, where available, that broadly describe the core features of LB dementia, i.e. motor impairment [Unified Parkinson’s Disease Rating Scale Part II (UPDRS III)], cognitive fluctuations, visual hallucinations (NPI_hall_ frequency × severity subscore) and sleep disturbance (NPI_sleep_ subscore). No significant associations were found for cognitive fluctuations or sleep; however, there were significant correlations with visual hallucinations and motor severity, i.e. NPI_hall_ with ^syn^PC_1_ (ρ = 0.49, *P* = 0.023)^*n* = 17^ (Fig. 4A) and UPDRS III with ^syn^PC_4_ (ρ = 0.49, *P* = 0.02)^*n* = 18^ (Fig. 4A) but not with ^tau^PC_1_, ^tau^PC_2_ or ^amyl^PC_1_ (NPI_hall_: ρ≤0.30, *P* ≥ 0.10; UPDRS III: ρ≤0.28, *P* ≥ 0.12). Although in a relatively small number of cases, this suggests the α-synuclein patterns could be pathological markers of specific LB dementia symptoms.

## Discussion

In summary, we present the first spatial mapping of quantitative HP-tau, amyloid-β and α-synuclein TMA pathology in healthy ageing and a range of neurodegenerative dementias (AD, DLB, _mixed_AD/DLB, PDD). The study identified seven patterns, i.e. ^tau^PC_1_, ^tau^PC_2_, ^amyl^PC_1_, ^syn^PC_1_, ^syn^PC_2_, ^syn^PC_3_ and ^syn^PC_4_, of pathological lesion distribution that could be described as groupings of strongly correlated regions of specific brain pathology, where distinct topographical scores of synuclein pathology were apparent both within and between DLB, _mixed_AD/DLB and PDD, that could explain some of the symptomatic variations and different spreading patterns. In LB dementia, correlations with parameters broadly representative of the core features, revealed associations with motor impairment and visual hallucinations within ^syn^PC_4_ and ^syn^PC_1_, respectively, which may suggest pathological signatures of the clinical phenotype.

Two patterns emerged from the HP-tau analyses (inferior temporal limbic, neocortical). As expected, both pattern scores were highest for AD and ^mixed^AD/DLB groups compared to DLB, PDD and controls. However, some degree of HP-tau burden was particularly associated with the inferior temporal limbic pattern in non-AD groups. These patterns appear to recapitulate Braak AD neuropathologic changes, which span ageing and dementia, i.e. inferior temporal limbic (Braak I to III) and neocortical (Braak IV to VI).^1^ For amyloid-β, a global pattern was identified, capturing most of the pathological spread according to Thal phase staging,^2^ i.e. neocortical → limbic → striatum → brainstem-cerebellum (latter not assessed in our study), with average pattern scores of AD > _mixed_AD/DLB > DLB > PDD > controls. There was also a degree of variation in the scores of aged healthy controls, which concurs with other reports that suggest as many as a third of healthy older adults show significant amyloid-β deposition.^32^ However, in the absence of HP-tau co-pathology this would be unlikely to have any clinical implications; certainly, an argument has been proffered that there is an element of age-associated amyloid-β accumulation that is clinically asymptomatic until sufficient aggregation of HP-tau is reached.^33^ For α-synuclein, four patterns emerged from the analyses, i.e. posterior temporal-occipital, frontal-anterior temporal, temporoparietal-insulo-cingulate, and frontostriatal-amygdala, with as expected, higher scores in the α-synucleinopathies. The patterns comprised of striatal and sub-elements of limbic and neocortical structures that broadly characterize aspects of pathological progression of α-synuclein according to McKeith.^26^ Despite most pattern scores conforming to their corresponding pathological classifications, large between-subject variations were observed within each dementia group, and are possible indicators of inter-individual disease/symptom heterogeneity. However, this theory would need to be tested in cohorts with sufficiently detailed clinical data. Quantifiable patterns of specific pathologies (present study) have the potential to offer new opportunities to investigate, on a continuous scale, proxies of pathological burden associated with selected topographies. These parameters, along with ante-mortem biomarker/clinical measures, could facilitate integrative models to provide a framework into the temporal relationship linking behavioural and related biomarkers during life with pathology, where likely outcomes would include predictors of symptomatic likelihood, disease trajectory and treatment response, thus enabling further targeted interventions for AD and LB dementias. Moreover, each group with significant LB pathology (DLB, _mixed_AD/DLB, PDD) demonstrated contrasting topographical pattern scores i.e. DLB > PDD in ^tau^PC_2_ and ^syn^PC_1_, DLB > _mixed_AD/DLB in ^syn^PC_1,4_, _mixed_AD/DLB > DLB and PDD in ^syn^PC_2,3_, as well as DLB and PDD > _mixed_AD/DLB in ^syn^PC_4_. This supports the view of higher prevalence of temporal-limbic tau pathology in DLB relative to PDD, and the idea of different proteinopathic spread patterns among the α-synucleinopathies.^34,35^ In particular, greater pattern scores of ^syn^PC_1,4_ in DLB relative to _mixed_AD/DLB, while greater scores of ^syn^PC_2,3_ in _mixed_AD/DLB compared to DLB is notable and suggests that DLB cases with significant AD co-pathology, warranting a mixed diagnosis, has a more ‘modulated’ α-synuclein spreading pattern than DLB cases without significant AD co-pathology, implying HP-tau and amyloid-β as pathologic moieties are individually or mutually affecting the pattern of pathological spread of α-synuclein in _mixed_AD/DLB compared to DLB. Further studies are required to reveal the possible modes of interaction among these pathologies in DLB with and without significant AD co-pathology. Given the above findings, scope for new biological staging systems in LB dementia, where patterns as well as multiple other co-existing pathologies, should be considered.

Relationships of pathological pattern scores with age at death, global cognition (MMSE) and global cognitive decline (ΔMMSE_avg_) were assessed collectively in dementia cases (AD, DLB, _mixed_AD/DLB, PDD). Age at death positively correlated with amyloid-β but negatively with α-synuclein. The former indicates age-related increases in amyloid-β burden, while the latter a marker of disease severity in the synucleinopathies, implying a possible shorter disease course as a consequence of increased α-synuclein deposition. Certainly, in LB dementia, α-synuclein burden is one of the strongest pathological predictors of a shorter interval between onset of motor and dementia symptoms and survival.^36^ MMSE closest to death was associated with both neocortical HP-tau and amyloid-β PC pattern scores in dementia cases and consistent with studies that have shown links between cognition and tau/amyloid burden in the dementias.^37-39^ In addition, a simple gauge of cognitive decline (ΔMMSE_avg_) was also found to correlate with inferior temporal limbic HP-tau and amyloid-β PC scores in dementia cases that agreed with previous reports of increased amyloid and medial temporal tau levels as predicting greater cognitive progression in ageing and dementia cohorts.^10,40-44^ This further reinforces the role of HP-tau and amyloid-β on cognition in AD and LB dementia.

Mediation analysis examined the inter-relationship between HP-tau and amyloid-β pattern scores on outcome measures of cognition in a combined dementia group (AD, DLB, _mixed_AD/DLB, PDD). It revealed that HP-tau and amyloid-β affect global cognition directly (HP-tau → MMSE) and indirectly (amyloid-β → HP-tau → MMSE), respectively, where the latter may imply a sequential interaction between amyloid and tau. This possibly coincides with the theory that amyloid-β offers an environment for HP-tau to accumulate, and why, for dementia cases (present study), high Braak tau stages were affiliated with high Thal phases (amyloid), i.e. both proteins are mutually inclusive for dementia. Symbiotic processes between tau and amyloid have been described in experimental models and human post-mortem tissue.^33,45^*In vitro* and *in vivo* studies suggest that there is a putative link between amyloid-β and tau; e.g. the addition of amyloid-β to cells expressing wild-type tau resulted in aggregations of tau in paired helical filaments,^46^ whilst injections of synthetic and brain-derived amyloid-β into tau transgenic mice resulted in increased tau aggregation in the injection site and synaptically connected brain regions, and induced tau seeding.^47-49^ Human and animal post-mortem studies have demonstrated co-localization of tau and amyloid-β in neurons and synapses.^50^ However, array tomography experiments reported that synpases were only positive for both amyloid-β and tau in a small percentage of cases (0.02%).^51^ There is evidence of conjunction effects between the two pathologies on decreased clinical function with ante-mortem PET imaging in specific brain regions.^52^ Mediation methods have been used to study relationships involving amyloid and tau, but only in AD. Specifically, Mungas *et al*.^53^ showed amyloid-β mediated the relationship between age and APOEε4 status on tau, while Bejanin *et al*.^19^ reported both direct and indirect effects of ^18^F-AV1451 uptake (tau) on cognitive performance mediated by grey matter volume. Furthermore, in a sub-sample of AD and LB dementia cases, there was a slight trend of a direct effect with ^tau^PC_2_ (temporal-limbic pattern) on cognitive progression. This seems logical, given the pattern captures the range of early tau neuropathologic spread (Braak I–III), which may be more sensitive to progression, and indeed implicates AD and LB dementia. The result aligns to some extent with previous ante-mortem imaging of greater baseline temporo-parietal tau PET binding as a predictor of cognitive decline in AD.^21,54^ Our findings, although tentative, emerged from quantitative pathological assessment and as such, may have stronger support for the administration of disease-modifying therapies that target amyloid-β and HP-tau across the spectrum of neurodegenerative dementias, and speak to the major push toward targeting amyloid-β in preclinical AD trials.^55^

Correlations in a merged α-synucleinopathy group (DLB, _mixed_AD/DLB, PDD) were performed between pathological pattern scores and pre-mortem measures broadly characterizing the core symptoms of LB dementia, i.e. parkinsonism, cognitive fluctuations, visual hallucinations and sleep disturbance. Significant associations of motor severity and visual hallucinations were identified within the α-synuclein frontostriatal-amygdala (^syn^PC_4_) and occipital-posterior temporal (^syn^PC_1_) patterns, respectively. Frontostriatal circuits mediate motor and cognitive functions, and receive inputs through dopaminergic, cholinergic, serotonergic and noradrenergic cell groups. The amygdala also receives afferent connections from the substantia nigra.^56^ Thus, the frontostriatal-amygdala pattern, and its association with motor severity, could signify LB burden and disruption of the mesocortical and nigrostriatal dopaminergic pathways. The neural circuitry of visual hallucinations in LB dementia is less clear and likely to involve multiple systems. Given the roles of both the occipital and inferior temporal cortices in visual perception and object recognition, it seems logical that these areas are then activated during visual hallucinations.^57^ The occipital-posterior temporal pattern, and its correlates with visual hallucination severity/frequency, may point to pathological lesions of the visual system in LB dementia. Indeed, in early pathological studies, higher Lewy body densities were observed within the neocortex^58^ and temporal lobe^59^ that associated with visual hallucinations in DLB. Interestingly, the pattern also contained hubs within the primary auditory cortex, which may have implications for auditory hallucinations, which often accompany the visual disturbances.^60^ There is also evidence of retinal/optic nerve α-synuclein deposition in the α-synucleinopathies,^61^ but direct associations of α-synuclein related retinal changes with visual hallucinations are tentative,^62^ while there is clearer indication that visual hallucinations are driven by distributed network changes, particularly in the brain visual system or higher cognitive systems.^63^ Colleagues have also studied the lateral geniculate nucleus in DLB with no obvious correlates.^64^ However, we acknowledge that α-synuclein changes elsewhere may have relevance to the areas examined and the clinical phenotype. In summary, the identified ‘patterns’ might be pathological markers of LB dementia symptomatology and could, after necessary testing/validation, be integrated into semiquantitative routine pathological assessment. Future repeated studies in larger LB dementia cohorts with well characterized ante-mortem clinical data will be required to verify the present results, and to possibly reveal other core symptomatic correlates in these conditions.

Although this is the first study to spatially map quantitative HP-tau, amyloid-β and α-synuclein pathology and establish associations with the cognitive and clinical phenotype in a large set of dementia cases, it was not without its limitations. First, all TMA data were obtained exclusively from the right hemisphere. Second, in theory, the 3 mm punch biopsies should have encompassed all cortical layers, capturing the full pathological development. However, though the TMA methodology allows the accurate assessment of pathological lesions in multiple brain regions, there is an inherent bias associated with this type of technique. It has been previously shown that densities of pathological protein aggregates (e.g. amyloid-β) differs between gyri and sulci,^65^ and as such we have tried to limit anatomical bias by sampling gyri and sulci of each cortical region within the TMA block. Other brain regions incorporated into the TMA block, such as the striatum and thalamus, are more complex structures containing multiple nuclei, and whilst this technique is aimed at providing an overview of pathology present, we suggest a more comprehensive sampling protocol when investigating individual brain regions. Third, we did not examine the substantia nigra, hippocampus, cerebellum or other critical structures, as this was considered an inefficient use of these tissues, as the TMA procedure would then preclude other researchers from requesting those blocks from the brain bank. Fourth, we studied diagnostically relevant pathologies related to AD and synucleinopathies but did not address the broader questions of how other pathologies, such as cerebral amyloid angiopathy (CAA), cerebrovascular disease and TDP-43, relate to the present findings. Fifth, available cases with ante-mortem measures of cognition were relatively small and thus may be underpowered for some analyses, particularly the mediation modelling. Sixth, although MMSE was used to assess cognitive function due to its ease of administration, other measures may be superior in assessing such symptoms in these individuals during life. Seventh, the calculation of cognitive progression assumed linearity since in the majority of cases with longitudinal data, only two ante-mortem assessments of MMSE were available. Eighth, the time between cognitive assessments during life and autopsy was varied (range 6.3 years) and may not accurately represent the degree of cognitive impairment at the time of death. Last, future directions would need to contemplate the laterality issue as well as consider other pathologically relevant proteins, such as CAA, TDP-43, pyroglutamylated amyloid and α-synuclein phosphorylation at serine 129.

In conclusion, a number of specific pathological lesion patterns were identified across a range of neurodegenerative dementias (AD, DLB, _mixed_AD/DLB, PDD). Distinct pattern scores of α-synuclein pathology were apparent in DLB, _mixed_AD/DLB and PDD, which could explain some of the disease heterogeneity and differing spreading patterns among these conditions. Visual hallucinations and motor severity were associated with distinct α-synuclein topographies in LB dementia that may be important to the clinical phenotype and could, after necessary testing/validation, be integrated into semiquantitative routine pathological assessment. Future studies involving larger AD and LB dementia cohorts with detailed clinical data will be required to confirm some of the present findings, and to establish whether relationships with pathology are generic or a function of specific groups across the neurodegenerative spectrum.

## Supplementary Material

awae372_Supplementary_Data

## Data Availability

The authors confirm that the data supporting the findings are available at the request of the senior author.
