# Supplementary material for: Patterns of tau, amyloid and synuclein pathology in ageing, Alzheimer’s disease and synucleinopathies
Source: Brain. 2024 Nov 12;148(5):1562–76. doi: 10.1093/brain/awae372 (PMC12073977; doi:10.1093/brain/awae372)
Supplement: awae372_Supplementary_Data [file awae372_supplementary_data.pdf]

Table S1. Punches (1-40), and approximated MNI coordinates and corresponding annotations.

Punch No	MNI coordinates			Annotation
1	46	41	31	Prefrontal (10/46)
2	39	41	28	Prefrontal (10/46)
3	29	41	20	Prefrontal (10/46)
4	30	41	10	Prefrontal white matter
5	33	26	55	Midfrontal (9)
6	31	26	46	Midfrontal (9)
7	25	26	38	Midfrontal (9)
8	26	26	21	Midfrontal white matter
9	4	20	42	Anterior cingulate (32)
10	3	20	27	Anterior cingulate (24)
11	16	-4	22	Caudate
12	30	-4	4	Putamen
13	21	-4	-1	Globus pallidus
14	40	-4	4	Anterior insula
15	23	-4	-19	Amygdala
16	28	-4	-40	Anterior fusiform
17	40	-10	65	Precentral gyrus (4)
18	43	-10	48	Precentral gyrus (4)
19	40	-10	9	Posterior insula
20	9	-10	7	Thalamus
21	19	-10	-32	Parahippocampal gyrus
22	24	-10	-36	Parahippocampal gyrus
23	30	-10	-33	Parahippocampal gyrus
24	31	-10	-42	Posterior fusiform
25	40	-10	-28	Posterior fusiform
26	62	-18	11	Transverse temporal gyrus (41,42)
27	67	-18	2	Superior temporal gyrus (22)
28	68	-18	-8	Middle temporal gyrus (21)
29	63	-18	-10	Middle temporal gyrus (21)
30	56	-18	-12	Middle temporal gyrus (21)
31	43	-50	60	Parietal (40)
32	29	-50	46	Parietal (40)
33	65	-50	20	Superior temporal gyrus (22)
34	50	-50	19	Superior temporal gyrus (22)
35	38	-50	12	Temporal white matter
36	25	-93	-8	Occipital (37/19)
37	13	-93	-12	Inferior occipital (17)
38	6	-93	-1	Superior occipital (17)
39	18	-93	5	Superior occipital (17)
40	21	-93	7	Occipital white matter

(Brodmann area).

Table S2. Truncated set of regions (1-23), approximated MNI coordinates and corresponding annotations.

Region No	MNI coordinates			Annotation	Figure 1 annotation
1	38	41	26	Prefrontal (10/46)	$PF^{10/46}$
2	29	26	46	Midfrontal (9)	MF^{9}
3	4	20	42	Anterior Cingulate (32)	AC^{32}
4	3	20	27	Anterior Cingulate (24)	AC^{24}
5	16	-4	22	Caudate	C
6	30	-4	4	Putamen	P
7	21	-4	-1	Globus pallidus	Gp
8	40	-4	4	Anterior insula	I_{ant}
9	23	-4	-19	Amygdala	Am
10	28	-4	-40	Anterior fusiform	F_{ant}
11	41	-10	56	Precentral gyrus (4)	Pre^{4}
12	40	-10	9	Posterior insula	I_{pos}
13	9	-10	7	Thalamus	Th
14	24	-10	-34	Parahippocampal gyrus	PHG
15	35	-10	-35	Posterior fusiform	F_{pos}
16	62	-18	11	Transverse temporal gyrus (41,42)	$TTG^{41/42}$
17	67	-18	2	Superior temporal gyrus (22)	STG_{ant}
18	62	-18	-10	Middle temporal gyrus (21)	MTG
19	36	-50	53	Parietal (40)	P^{40}
20	57	-50	19	Superior temporal gyrus (22)	STG_{pos}
21	25	-93	-8	Occipital (37/19)	$O^{37/19}$
22	13	-93	-12	Inferior occipital (17)	$O^{17}{}_{inf}$
23	12	-93	2	Superior occipital (17)	$O^{17}{}_{sup}$

(Brodmann area).

Table S3. Percentage area of the tissue covered by tau, β-amyloid and α-synuclein immunopositivity within each TMA region across all ageing and dementia cases.

Region No	Figure 1 annotation	$Tau^{n=140}$ (% area)	$Amyloid^{n=132}$ (% area)	$Synuclein^{n=158}$ (% area)
1	$PF^{10/46}$	0.038 ± 0.008	0.072 ± 0.007	0.0004 ± 0.0001
2	MF^{9}	0.048 ± 0.009	0.075 ± 0.007	0.0007 ± 0.0002
3	AC^{32}	0.040 ± 0.009	0.061 ± 0.008	0.003 ± 0.001
4	AC^{24}	0.041 ± 0.008	0.052 ± 0.006	0.002 ± 0.0009
5	C	0.018 ± 0.005	0.041 ± 0.005	0.0006 ± 0.0001
6	P	0.011 ± 0.005	0.032 ± 0.004	0.001 ± 0.0002
7	Gp	0.012 ± 0.005	0.012 ± 0.002	0.0003 ± 0.00006
8	I_{ant}	0.090 ± 0.013	0.047 ± 0.005	0.004 ± 0.001
9	Am	0.140 ± 0.016	0.026 ± 0.003	0.005 ± 0.0009
10	F_{ant}	0.190 ± 0.020	0.036 ± 0.004	0.006 ± 0.002
11	Pre^{4}	0.038 ± 0.009	0.040 ± 0.004	0.0003 ± 0.00007
12	I_{pos}	0.067 ± 0.011	0.047 ± 0.005	0.002 ± 0.0007
13	Th	0.008 ± 0.003	0.027 ± 0.004	0.0003 ± 0.00007
14	PHG	0.150 ± 0.015	0.039 ± 0.004	0.002 ± 0.0008
15	F_{pos}	0.180 ± 0.019	0.048 ± 0.004	0.002 ± 0.0006
16	$TTG^{41/42}$	0.063 ± 0.011	0.049 ± 0.005	0.0004 ± 0.0001
17	STG_{ant}	0.068 ± 0.013	0.051 ± 0.005	0.0005 ± 0.0002
18	MTG	0.081 ± 0.013	0.058 ± 0.006	0.0007 ± 0.0002
19	P^{40}	0.069 ± 0.013	0.062 ± 0.006	0.0004 ± 0.0002
20	STG_{pos}	0.060 ± 0.012	0.058 ± 0.006	0.0004 ± 0.00009
21	$O^{37/19}$	0.054 ± 0.011	0.034 ± 0.003	0.0002 ± 0.00009
22	O^{17}_{inf}	0.055 ± 0.012	0.033 ± 0.004	0.0002 ± 0.00009
23	O^{17}_{sup}	0.060 ± 0.011	0.044 ± 0.005	0.0003 ± 0.0001

Values represent mean ± SEM.

Table S4. Extracted PCs and their loadings from PCA with direct oblimin rotation from individual tau, β-amyloid and α-synuclein TMA data in ageing and dementia.

Region	(n=140) $^{tau}PC_1$	$^{tau}PC_2$	(n=132) $^{amyl}PC_1$	$^{syn}PC_1$	(n=158) $^{syn}PC_2$	$^{syn}PC_3$	$^{syn}PC_4$
$PF^{10/46}$	**0.88**	0.02	**0.93**	0.17	0.23	-0.06	**0.63**
MF^{9}	**0.86**	0.06	**0.91**	0.14	**0.63**	-0.07	0.33
AC^{32}	**0.89**	-0.04	**0.88**	-0.08	0.05	**0.75**	0.17
AC^{24}	**0.82**	0.03	**0.82**	0.04	-0.01	**0.94**	0.07
C	**0.72**	-0.08	**0.90**	0.05	-0.04	0.36	**0.77**
P	0.17	0.03	**0.85**	-0.17	-0.001	0.16	**0.78**
Gp	0.41	-0.21	0.44	0.007	0.04	0.05	**0.77**
I_{ant}	0.47	0.50	**0.86**	-0.09	**0.92**	0.13	0.01
Am	-0.07	**0.79**	**0.68**	-0.07	-0.004	0.06	**0.64**
F_{ant}	0.18	**0.80**	**0.80**	-0.09	**0.89**	0.03	0.04
Pre^{4}	**0.75**	0.01	**0.79**	0.48	0.09	0.06	0.50
I_{pos}	**0.62**	0.32	**0.88**	0.002	-0.02	**0.93**	0.13
Th	0.29	-0.14	**0.72**	0.44	0.08	0.02	0.55
PHG	0.20	**0.78**	**0.83**	-0.05	**1.02**	0.03	-0.27
F_{pos}	0.27	**0.78**	**0.88**	-0.003	**0.77**	-0.10	0.14
$TTG^{41/42}$	**0.61**	0.37	**0.89**	**0.61**	0.11	-0.25	0.49
STG_{ant}	**0.72**	0.25	**0.86**	0.28	0.10	**0.81**	-0.03
MTG	**0.80**	0.25	**0.94**	0.13	**0.83**	0.22	-0.02
P^{40}	**0.84**	0.12	**0.85**	0.08	0.26	**0.85**	-0.009
STG_{pos}	**0.79**	0.20	**0.92**	**0.82**	-0.03	0.07	0.16
$O^{37/19}$	**0.79**	0.05	**0.85**	**0.98**	-0.008	-0.003	-0.14
O^{17}_{inf}	**0.77**	0.18	**0.81**	**0.94**	-0.02	0.12	-0.12
O^{17}_{sup}	**0.71**	0.14	**0.83**	**0.76**	-0.07	0.48	-0.09
$Eigenvalue_{initial}$	12.2	2.0	16.1	10.1	3.5	2.5	2.1
$Eigenvalue_{rotated}$	11.3	5.6	Na	5.9	6.1	6.4	6.1
Variance %	53.0	8.9	70.1	44.0	15.3	10.8	8.9

Loadings significantly contributing to the PC are shown in **bold** (≥ |0.60|).
Na = Not applicable.

Table S5. Pathological PC pattern scores for each diagnostic group stratified by, where applicable, APOE4 status (absent, present).

	Controls	AD	DLB	^{mixed}AD/DLB	PDD	Statistic, p-value[a]
Tau PC_1^{absent}	-0.44 ± 0.04	0.19 ± 1.19	-0.46 ± 0.11	0.38 ± 0.49	-0.46 ± 0.03	|ρ| ≤ 0.35, p ≥ 0.09
Tau $PC_1^{present}$	-0.49 ± 0.06	0.92 ± 1.17	-0.01 ± 1.44	0.41 ± 0.91	-0.54 ± 0.13	
Tau PC_2^{absent}	-0.64 ± 0.35	0.11 ± 0.99	-0.29 ± 0.69	1.18 ± 0.96	-0.58 ± 0.37	|ρ| ≤ 0.42, p ≥ 0.18
Tau $PC_2^{present}$	-0.32 ± 0.54	0.29 ± 1.11	0.20 ± 1.21	0.60 ± 0.74	0.16 ± 1.22	
Amyl PC_1^{absent}	-0.75 ± 0.35	0.33 ± 0.76	-0.56 ± 0.29	0.36 ± 0.15	-0.71 ± 0.41	|ρ| ≤ 0.68, p ≥ 0.10
Amyl $PC_1^{present}$	-0.53 ± 0.63	0.90 ± 0.75	0.30 ± 1.21	0.59 ± 1.08	-0.10 ± 0.59	
Syn PC_1^{absent}	-0.24 ± 0.001	-0.24 ± 0.0002	0.73 ± 1.17	-0.27 ± 0.02	0.33 ± 0.82	|ρ| ≤ 0.26, p ≥ 0.15
Syn $PC_1^{present}$	-0.24 ± 0.0005	-0.25 ± 0.04	1.38 ± 3.05	-0.005 ± 0.64	-0.02 ± 0.39	
Syn PC_2^{absent}	-0.26 ± 0.001	-0.26 ± 0.001	0.74 ± 1.50	-0.18 ± 0.08	0.008 ± 0.29	|ρ| ≤ 0.45, p ≥ 0.12
Syn $PC_2^{present}$	-0.26 ± 0.0005	-0.24 ± 0.09	0.08 ± 0.32	0.78 ± 2.47	0.04 ± 0.74	
Syn PC_3^{absent}	-0.17 ± 0.001	-0.17 ± 0.0001	0.41 ± 0.74	-0.17 ± 0.02	-0.05 ± 0.14	|ρ| ≤ 0.33, p ≥ 0.26
Syn $PC_3^{present}$	-0.17 ± 0.0004	-0.18 ± 0.006	0.12 ± 0.16	0.04 ± 0.30	0.03 ± 0.35	
Syn PC_4^{absent}	-0.43 ± 0.006	-0.43 ± 0.002	1.57 ± 2.09	-0.38 ± 0.04	0.61 ± 1.02	|ρ| ≤ 0.26, p ≥ 0.33
Syn $PC_4^{present}$	-0.43 ± 0.0004	-0.39 ± 0.15	0.47 ± 0.88	0.36 ± 1.37	0.69 ± 1.66	

Values denote (mean ± SD).

Tau PCs (absent, present): Controls (25, 4); AD (10, 15); DLB (7, 10); $_{mixed}$AD/DLB (3, 9); PDD (6, 6).
Amyloid PC (absent, present): Controls (25, 5); AD (8, 15); DLB (7, 7); $_{mixed}$AD/DLB (3, 11); PDD (5, 4).
Synuclein PCs (absent, present): Controls (25, 8); AD (13, 19); DLB (7, 9); $_{mixed}$AD/DLB (3, 11); PDD (6, 7).

[a]For each pathological PC score, Spearman correlations for individual groups were performed for associations with APOE4 status (absent, present).
